# Tumor regression grade combined with post‐therapy lymph node status: A novel independent prognostic factor for patients treated with neoadjuvant therapy followed by surgery in locally advanced gastroesophageal junction and gastric carcinoma

**DOI:** 10.1002/cam4.6597

**Published:** 2023-09-25

**Authors:** Hongyan Yin, Qian Yao, Yi Xie, Dongfeng Niu, Wenya Jiang, Huiying Cao, Xujiao Feng, Yanyan Li, Yilin Li, Xiaotian Zhang, Lin Shen, Yang Chen

**Affiliations:** ^1^ Department of Gastrointestinal Oncology, Key Laboratory of Carcinogenesis and Translational Research (Ministry of Education) Peking University Cancer Hospital and Institute Beijing China; ^2^ Department of Gastroenterology Cangzhou People's Hospital Cangzhou China; ^3^ Department of Pathology, Key Laboratory of Carcinogenesis and Translational Research (Ministry of Education) Peking University Cancer Hospital and Institute Beijing China

**Keywords:** gastroesophageal junction and gastric carcinoma, immunotherapy, lymph node status, neoadjuvant therapy, tumor regression grade

## Abstract

**Background:**

Tumor regression grade (TRG) is a measure of histopathological response to neoadjuvant therapy (NAT). Post‐therapy lymph node (ypN) metastasis was reported as a prognostic factor. However, the evaluation of the treatment effectiveness of NAT has not been well studied. Here, we explored whether TRG combined with ypN status could be a prognostic factor for gastroesophageal junction (GEJ) and gastric cancer (GC). Besides, we aimed at making clear the association of different neoadjuvant regimens with different TRG and ypN status.

**Methods:**

376 patients with GEJ or GC accepting NAT in Peking University Cancer Hospital were retrospectively collected from January 1, 2003 to June 30, 2021. According to TRG and ypN status, patients were innovatively categorized into four groups: TRG0N0, TRG1‐3N0, TRG0‐1N+, and TRG2‐3N+. We applied Kaplan–Meier method and log‐rank test to testify the differences in disease free survival (DFS) and overall survival (OS) among four groups. Univariate and multivariate analyses were performed to examine the relationships between TRG combined with ypN status and prognosis.

**Results:**

We observed significant survival differences among the four groups (*p* < 0.001, respectively). Median DFS and OS of patients with TRG0N0, TRG1‐3N0, and TRG0‐1N+ were not reached, whereas these of patients with TRG2‐3N+ were 17.37 months (95% CI, 14.14–20.60 months) and 39.97 months (95% CI, 27.05–52.89 months). TRG combined with ypN status was still an independent predictor for both DFS (*p* < 0.001) and OS (*p* < 0.001) in multivariate analysis. Chi‐squared test showed TRG combined with ypN status was significantly associated with different preoperative treatments (*p* < 0.001). Patients receiving immunotherapy achieved the highest TRG0N0 rate (31.9%).

**Conclusion:**

Our results demonstrate that TRG combined with ypN status is a novel independent predictor of both DFS and OS in resectable, locally advanced GEJ and GC. Neoadjuvant immunotherapy achieved the highest TRG0N0 rate.

## Graphical abstract

Whether tumor regression grade (TRG) combined with ypN status could be a prognostic factor for gastroesophageal junction (GEJ) and gastric cancer (GC) was unclear. In our study, we retrospectively analyzed 376 patients’ data to elucidate the relationships between TRG combined with ypN status and prognosis. These patients were innovatively categorized into four groups: TRG0N0, TRG1‐3N0, TRG0‐1N+, and TRG2‐3N+. Then we evaluated the predictive value of TRG combined wiht ypN status. Our results suggest that TRG combined with ypN status is a novel independent predictor of both DFS and OS in resectable, locally advanced GEJ, and GC. The application of neoadjuvant immunotherapy significantly increased the TRG0N0 rate, which represented the best prognosis in our findings.

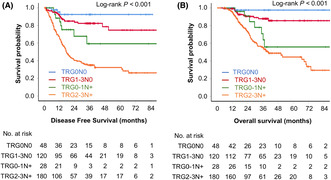

## 1 INTRODUCTION

Gastric cancer (GC) is the fourth leading cause of cancer‐related mortality worldwide and remains a significant global health threat.^1^ Patients in high‐incidence regions are usually diagnosed with GC at the advanced stage. the MAGIC and FNCLCC/FFCD studies proved that neoadjuvant therapy (NAT) significantly improved the survival of patients with locally advanced GC.^2^, ^3^, ^4^ RESOLVE study further laid the foundation of NAT followed by D2 gastrectomy for GC.^5^ NAT has become a standard treatment for resectable gastroesophageal junction (GEJ) and GC based on the National Comprehensive Cancer Network (NCCN) and European Society for Medical Oncology (ESMO) guidelines,^6^, ^7^ as well as Chinese Society of Clinical Oncology (CSCO) guidelines.^8^ Despite receiving standard treatments, half of the patients face the risk of tumor recurrence and death of carcinoma. Thus, predicting the long‐term prognosis of patients receiving NAT is an urgent need.

To date, no prognostic factor has filled the predictive vacancy for patients undergoing NAT and radical gastrectomy. The American Joint Committee on Cancer (AJCC)/Union for International Cancer Control (UICC) proposed neoadjuvant therapy TNM staging (ypTNM) as a prognostic marker for patients accepting NAT. Unfortunately, patients who achieve pCR or ypT0 with post‐therapy lymph node (ypN) positive after NAT are excluded from the ypTNM system for GC, which makes it incomplete. Tumor regression grade (TRG) is defined as the pathological evaluation of tumors resected after NAT to perform a quantitative analysis of the therapeutic effect on tumors. Notably, ypN status is also a response to NAT. Both TRG and ypN were previously examined in esophageal squamous cell carcinoma, gastric, and rectal cancer.^9^, ^10^, ^11^, ^12^, ^13^, ^14^, ^15^, ^16^ According to a few studies, ypN status instead of TRG is an independent prognostic factor for GEJ and GC.^10^, ^13^, ^14^ However, these studies analyzed the prognostic value of ypN status and TRG separately, which is not sufficient. Dichotomizing patients into ypN‐negative (ypN0) and ypN‐positive (ypN+) groups is also far from enough. By achieving pathological complete response (pCR), being TRG 0 and ypN0, patients can gain a better outcome than those who do not achieve pCR.^17^ From a study by Smyth, we can find that the overall survival (OS) of patients with Mandard TRG 1–2 in the ypN+ group was longer than that of patients with Mandard TRG 3–5 in GC.^10^ Researches precisely assessing the prognosis after NAT are thus warranted. Furthermore, both TRG and ypN status could reflect the effectiveness of preoperative treatment; however, whether different neoadjuvant regimens can generate different levels of TRG combined with ypN status have not been determined.

In this study, we aimed to evaluate the clinical outcomes of patients receiving NAT, so as to provide surrogate endpoints for NAT in clinical trials. We focused on creating a novel prognostic factor and highlighted its predictive value. Additionally, we opted to expose whether preoperative immunotherapy could improve TRG and ypN status.

## 2 MATERIALS AND METHODS

### 2.1 Patients

Patients diagnosed with locally advanced GEJ or GC and administered NAT at Peking University Cancer Hospital between January 1, 2003 and June 30, 2021 were selected for this study. The inclusion criteria were: (1) patients pathologically diagnosed with GEJ or gastric adenocarcinoma before NAT; (2) patients with locally advanced stage of the disease (8th AJCC clinical stage III–IVA); (3) patients administered at least two cycles of preoperative treatment, including chemotherapy, anti‐HER‐2 therapy, and ICI therapy; and (4) patients who underwent D2 gastrectomy surgery and R0 resection. The exclusion criteria were: (1) patients with concurrent malignant tumors; (2) patients treated with radiotherapy; (3) patients subjected to D1 gastrectomy or R1/R2 surgical resection; and (4) patients whose TRG was unavailable. Finally, 376 of the 723 patients were enrolled in the study.

### 2.2 Neoadjuvant therapy regimens

Patients received the following NAT regimens: immunotherapy (69 patients; the patients did not participate in clinical trials); platin‐based doublet regimens (193 patients); taxol‐based or taxol‐platin‐based triplet regimens (94 patients); and anti‐HER‐2 therapy without immune checkpoint inhibitors (ICI) (20 patients). Written informed consent was obtained from patients or their legal guardians before treatment initiation.

### 2.3 TRG assessment and histopathological characters

TRG was assigned according to the NCCN standard. NCCN TRG was categorized into four groups as follows: TRG 0 (complete response, including lymph nodes): absence of viable cancer cells; TRG 1 (near‐complete response): presence of single cells or few small groups of cancer cells; TRG 2 (partial response): presence of residual cancer cells with evident tumor regression but a larger number of single cells or groups of cancer cells; and TRG 3 (poor or no response): presence of extensive residual cancer without evident tumor regression.^8^ TRG of the 376 participants were reviewed by two experienced gastrointestinal pathologists, respectively, and the numbers of regional lymph node metastases were determined by them. Histological types, degrees of cell differentiation, tumor sizes, post‐therapy infiltration depth (ypT), vascular or lymphatic invasion (LVI), and venous invasion (VI) were recorded in the patient's pathological report. Expression levels of human epidermal growth factor receptor 2 (HER‐2), epidermal growth factor receptor (EGFR), Epstein–Barr encoding region (EBER) and MMR status were detected via immunohistochemistry (IHC).

### 2.4 Follow‐up

Follow‐up was performed every 3 months during the first 2 years, every 6 months from the third year to the fifth year after surgery, and once per year thereafter. If patients had symptoms or signs of recurrence or metastasis, follow‐up visits were scheduled.

### 2.5 Statistical methods

Disease free survival (DFS) was calculated from the date of surgery to first tumor recurrence or death. OS was defined as the time from initial NAT to death from any cause or the last date of follow‐up. We defined different groups based on different TRG categories and ypN status. Differences in DFS and OS stratified by different combinations were evaluated implementing the Kaplan–Meier method and compared using the log‐rank test. A two‐tailed *p* < 0.05 was considered statistically significant. The four‐group combination was proven to be a favorable evaluation system. Univariate and multivariate analyses were performed using Cox regression and proportional hazard models. Univariate analysis comprising age, sex, completeness of local resection, TRG combined with ypN status, ypT stage, LVI, and VI was performed to determine the variable that was statistically correlated with prognosis. Statistically significant variables (*p* < 0.05) in univariate analysis were incorporated into the multivariate Cox regression models to analyze the survival difference among different groups. Neoadjuvant regimens among the four groups were compared using the chi‐squared test. SPSS software (version 26) was used to perform statistical analysis.

## 3 RESULTS

### 3.1 Patients

A total of 376 patients were enrolled in this study. The baseline characteristics of patients are listed in Table 1. The following TRG results were obtained for the enrolled patients: TRG 0 (51 cases, 13.56%), TRG 1 (52 cases, 13.83%), TRG 2 (173 cases, 46.01%), and TRG 3 (100 cases, 26.60%). The results for the ypN status of these patients were as follows: ypN0 (169 cases, 44.95%), ypN1 (86 cases, 22.87%), ypN2 (61 cases, 16.22%), and ypN3 (60 cases, 15.96%). Forty‐eight (12.77%) patients achieved pCR.

**TABLE 1:** Patient characteristics.

Characteristics	*N* (%)
Age (years)	
≤65	268 (71.28)
>65	108 (28.72)
Sex	
Female	88 (23.40)
Male	288 (76.60)
Location of tumor	
Gastroesophageal junction	150 (39.89)
Stomach	226 (60.11)
Grade of differentiation (pretreatment)	
Well	9 (2.39)
Moderate	116 (30.85)
Poor	235 (62.50)
Unknown	16 (4.26)
Lauren classification (pretreatment)	
Intestinal	179 (47.61)
Diffuse	87 (23.14)
Mixed	78 (20.74)
Unknown	32 (8.51)
Clinical T stage	
T2	2 (0.53)
T3	105 (27.93)
T4	257 (68.35)
Tx	12 (1.19)
Clinical *N* status	
Negative	20 (5.32)
Positive	326 (86.70)
Unknown	30 (7.98)
Neoadjuvant regimens	
ICI‐containing regimens	69 (18.35)
Platin‐based doublet regimens	193 (51.33)
Taxol‐based double or triplet regimens	94 (25.00)
Anti‐HER‐2 regimens	20 (5.32)
Histologic type (post‐treatment)	
Adenocarcinoma	317 (84.31)
Adenosquamous carcinoma	4 (1.06)
Hepatic adenocarcinoma	7 (1.86)
No tumor cells	48 (12.77)
Grade of differentiation (post‐treatment)	
Well	3 (0.80)
Moderate	83 (22.07)
Poor	199 (52.93)
No tumor cells	48 (12.77)
Unknown	43 (11.44)
Lauren classification (post‐treatment)	
Intestinal	130 (34.57)
Diffuse	64 (17.02)
Mixed	70 (18.62)
Unknown	64 (17.02)
No tumor cells	48 (12.77)
ypT stage	
ypT0	51 (13.56)
ypT1	28 (7.45)
ypT2	56 (14.90)
ypT3	177 (47.07)
ypT4	64 (17.02)
ypN stage	
ypN0	169 (44.95)
ypN1	86 (22.88)
ypN2	61 (16.22)
ypN3	60 (15.96)
TRG stage	
0	51 (13.56)
1	52 (13.83)
2	173 (46.01)
3	100 (26.60)
Vascular or lymphatic invasion	
No	231 (61.44)
Yes	144 (38.30)
Unknown	1 (0.27)
Venous invasion	
No	194 (51.60)
Yes	177 (47.07)
Unknown	5 (1.33)
HER‐2 expression	
0	195 (51.86)
1+	78 (20.74)
2+	49 (13.03)
3+	33 (8.78)
Unknown	21 (5.59)
EGFR expression	
0	38 (10.11)
1+	89 (23.67)
2+	122 (32.45)
3+	78 (20.74)
Unknown	49 (13.03)
MMR status	
MMR‐deficient	25 (6.65)
MMR‐proficient	288 (76.60)
Unknown	63 (16.76)
EBER expression	
Positive	19 (5.05)
Negative	297 (78.99)
Unknown	60 (15.96)

Abbreviations: EBER, Epstein–Barr encoding region; EGFR, epidermal growth factor receptor; HER‐2, human epidermal growth factor receptor 2; ICI, immune checkpoint inhibitors; MMR, mismatch repair; TRG, tumor regression grade; ypN, post‐therapy lymph node; ypT, post‐therapy infiltration depth.

### 3.2 TRG combined with ypN status is associated with DFS and OS

The median follow‐up time was 38.50 months (95% CI, 37.49–39.51 months). The median DFS (mDFS) and median OS (mOS) for the entire population were not achieved. However, 58.1% of the population achieved the 3‐year DFS while 71.4% achieved the 3‐year OS.

First, we divided patients according to their TRG levels (Figure S1A,B). The mDFS for patients with TRG 0 and TRG 1 had not reached, while those for patients with TRG 2 and TRG 3 were 45.83 months (95% CI, not reached, *p* < 0.001) and 30.83 months (95% CI, 14.23–47.44 months, *p* < 0.001), respectively. The mOS for patients with TRG 3 was 68.43 months (95% CI, 49.57–87.29 months, *p* < 0.001). Notably, patients with the other TRG levels did not achieve the mOS. The 3‐year DFS for patients with TRG 0 was 90.8% compared with 78.8% for TRG 1, 51.5% for TRG 2, and 46.3% for TRG 3. Further, the 3‐year OS for patients with TRG 0 was 95.4% versus 87.5%, 66.2%, and 63.7% for TRG 1‐3, respectively. [Correction added on October 18, 2023 after first online publication. The values in the previous sentence have been updated in this version.]

Second, patients with ypN0 were compared to those with ypN+ (Figure S2A,B). Patients with ypN0 had not yet achieved the mDFS and mOS, whereas those with ypN+ had an mDFS of 19.20 months (95% CI, 14.71–23.69 months, *p* < 0.001) and an mOS of 40.53 months (95% CI, 19.97–61.09 months, *p* < 0.001). The 3‐year DFS was 85.1% and 38.1% for patients with ypN0 and ypN+, and the 3‐year OS was 90.2% and 56.9%, respectively.

Third, we dichotomized patients into the following two groups for the subsequent study: with or without pCR. We found that patients who achieved pCR demonstrated significantly better survival rates in terms of both DFS (*p* < 0.001) and OS (*p* < 0.001), compared to those who did not achieve pCR. (Figure S3A,B). The pCR group had not achieved the mDFS and mOS. In contrast, the non‐pCR group had an mDFS of 50.63 months (95% CI, 26.72–74.54 months) and mOS of 73.80 months (95% CI, not reached). The 3‐year DFS rates of the pCR group versus non‐pCR group were 92.5% versus 53.8% and the 3‐year OS rates were 97.6% versus 68.2%, respectively.

**FIGURE 1:**
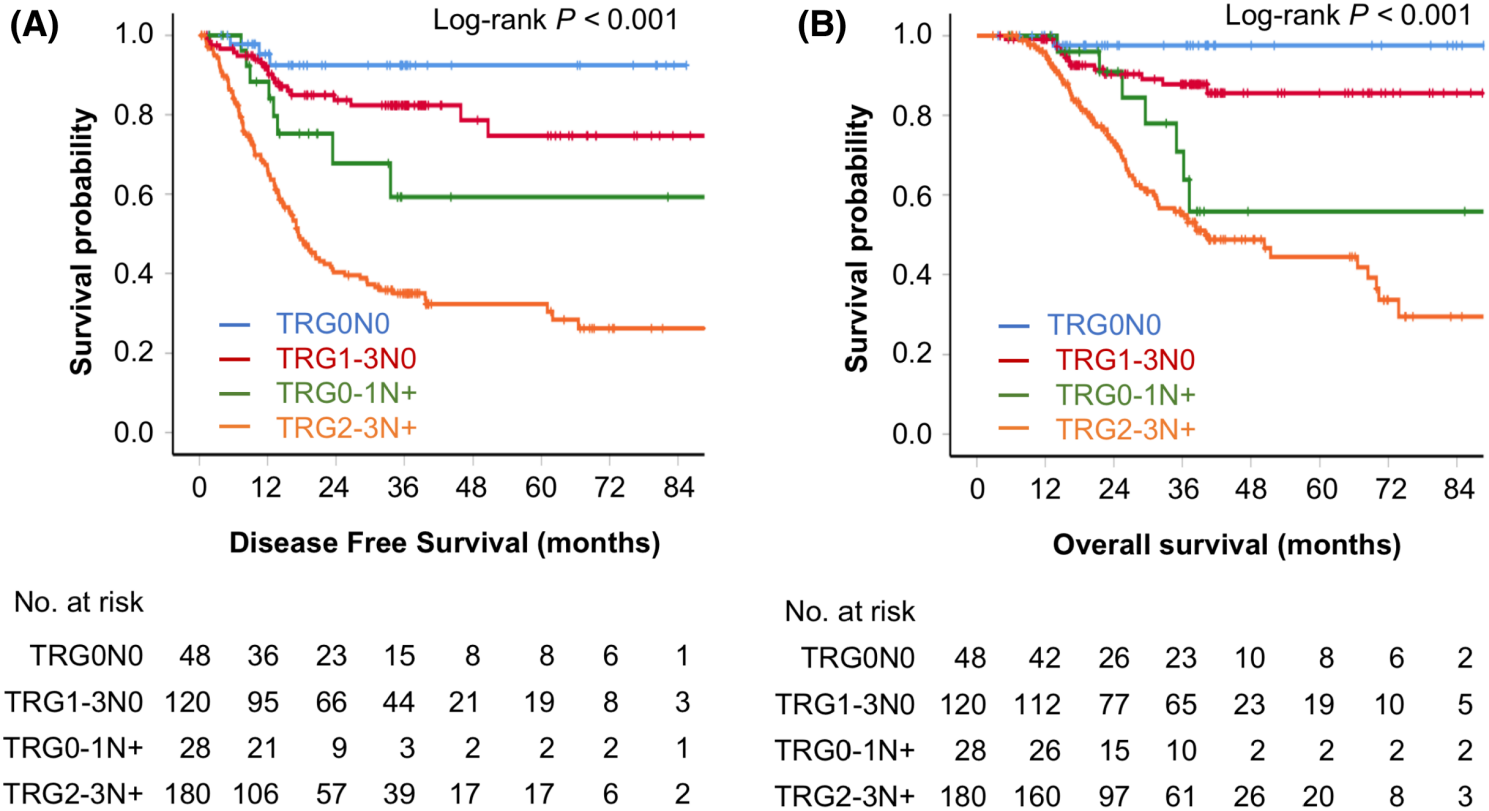
Differences of disease free survival (A) and overall survival (B) for patients separated into four groups by tumor regression grade (TRG) and post‐therapy lymph node (ypN) status: TRG0N0, TRG1‐3N0, TRG0‐1N+, and TRG2‐3N+ groups.

TRG and ypN status were assessed as two independent markers earlier. However, to perform a more precise distinction of patients with different survivals based on the different TRG categories combined with ypN status, patients were subdivided into the following eight groups: TRG0N0, TRG0N+, TRG1N0, TRG1N+, TRG2N0, TRG2N+, TRG3N0, TRG3N+ groups (Figure S4A,B). Groups with similar prognosis were combined based on survival curves. Thereafter, we split patients into four groups: TRG0N0, TRG1‐3N0, TRG0‐1N+, and TRG2‐3N+. Significant discriminations based on DFS (*p* < 0.001) and OS (*p* < 0.001) were found among the four groups as shown in Figure 1A,B. the mDFS and mOS of the TRG2‐3N+ group were 17.37 months (95% CI, 14.14–20.60 months) and 39.97 months (95% CI, 27.05–52.89 months), while those of other groups had not reached. Notably, 92.5% of patients in the TRG0N0 group achieved the 3‐year DFS while 82.4%, 59.5%, and 35.1% of patients in the TRG1‐3N0, TRG0‐1N+, and TRG2‐3N+ groups, respectively, achieved the 3‐year DFS. Further, 97.6% of patients in the TRG0N0 group achieved the 3‐year OS while 87.8%, 70.9%, and 54.9% of patients in the TRG1‐3N0, TRG0‐1N+, and TRG2‐3N+ groups, respectively, achieved the 3‐year OS.

### 3.3 TRG combined with ypN status is an independent prognostic factor based on multivariate analysis

We conducted univariate analysis to evaluate the prognostic value of age, sex, location of tumor, Lauren classification, ypT stage, LVI, and VI, TRG combined with ypN status, and neoadjuvant regimens on DFS and OS (Table 2). TRG combined with ypN status, ypT stage, LVI, and VI were all considered as prognostic predictors of DFS (*p* < 0.001, respectively) and OS (*p* < 0.001, respectively). Location of tumor and neoadjuvant regimens were only significantly associated with DFS (*p* = 0.019, *p* = 0.007, respectively), and not with OS (*p* = 0.12, *p* = 0.12, respectively). The statistically significant factors in the univariate analysis were included in the multivariate analysis (Table 3). TRG combined with ypN status was the strongest independently predictor for DFS. The hazard ratio (HR) for the TRG1‐3N0 group was 1.62 (95% CI, 0.45–5.86, *p* = 0.47), while that for the TRG0‐1N+ group was 3.01 (95% CI, 0.75–12.18, *p* = 0.12), and that for TRG2‐3N+ group was 5.70 (95% CI, 1.57–20.73, *p* = 0.008) compared with the TRG0N0 group. A similar trend was also observed for OS. The HR for TRG1‐3N0 group was 3.31 (95% CI, 0.41–26.52, *p* = 0.26), while that for TRG0‐1N+ group was 9.69 (95% CI, 1.15–81.98, *p* = 0.037), and that for TRG2‐3N+ group was 12.13 (95% CI, 1.52–96.88, *p* = 0.019) compared with TRG0N0 group. ypT3‐T4 and LVI‐positive led to significant deterioration in DFS only (ypT3‐T4: HR, 1.99; 95% CI, 1.09–3.45; *p* = 0.025; LVI‐positive: HR, 1.48; 95% CI, 1.01–2.18; *p* = 0.046; respectively). No other variables were confirmed to be independent surrogate predictors in this study.

**TABLE 2:** Univariate analysis of the factors affecting the DFS and OS of patients with gastroesophageal junction and gastric cancer and treated with neoadjuvant therapy.

		DFS (*N* = 376)			OS (*N* = 376)		
Variables	No. of cases	No. of events	HR (95% CI)	*P*	No. of events	HR (95% CI)	*p*
Age (years)							
≤65	268	99			70		
>65	108	41	1.04 (0.72–1.50)	0.84	28	1.01 (0.65–1.57)	0.95
Sex							
Female	88	31			24		
Male	288	109	0.89 (0.60–1.32)	0.56	74	1.04 (0.66–1.65)	0.87
Location of tumor							
Gastroesophageal junction	150	70			48		
Stomach	226	70	0.67 (0.48–0.94)	0.019	50	0.73 (0.49–1.08)	0.12
Lauren classification							
Intestinal	179	67		0.86	43		0.94
Diffuse	87	32	0.84 (0.55–1.28)	0.41	26	1.11 (0.68–1.81)	0.67
Mixed	78	30	0.96 (0.63–1.48)	0.86	20	1.08 (0.63–1.83)	0.78
Unknown	32	11	1.02 (0.54–1.93)	0.96	9	1.23 (0.60–2.52)	0.58
ypT stage							
ypT0‐T2	135	18			14		
ypT3‐T4	241	122	4.31 (2.63–7.08)	<0.001	84	3.59 (2.04–6.32)	<0.001
Vascular or lymphatic invasion							
No	231	55			37		
Yes	145	85	2.88 (2.05–4.04)	<0.001	61	2.82 (1.87–4.24)	<0.001
Venous invasion							
No	196	50			30		
Yes	180	90	2.05 (1.45–2.89)	<0.001	68	2.54 (1.65–3.90)	<0.001
Groups							
TRG0N0	48	3		<0.001	1		<0.001
TRG1‐3N0	120	20	2.56 (0.76–8.62)	0.13	13	4.81 (0.63–36.76)	0.13
TRG0‐1N+	28	8	5.17 (1.37–19.49)	0.015	7	13.41 (1.65–109.12)	0.015
TRG2‐3N+	180	109	12.42(3.94–39.13)	<0.001	77	23.38 (3.25–168.19)	0.002
Neoadjuvant regimens							
ICI‐containing regimens	69	12		0.007	5		0.12
Platin‐based doublet regimens	193	79	1.46 (0.79–2.70)	0.23	63	1.80 (0.71–4.52)	0.21
Taxol‐based double or triplet regimens	94	47	2.22 (1.18–4.20)	0.014	29	2.22 (0.85–5.78)	0.10
Anti‐HER‐2 regimens	20	2	0.40 (0.089–1.78)	0.28	1	0.33 (0.038–2.79)	0.31

Abbreviations: CI, confidence interval; DFS, disease free survival; HER‐2, human epidermal growth factor receptor 2; HR, hazard ratio; ICI, immune checkpoint inhibitors; OS, overall survival; TRG, tumor regression grade; ypT, post‐therapy infiltration depth.

**TABLE 3:** Multivariate analysis of the factors affecting the DFS and OS of patients with gastroesophageal junction and gastric cancer and treated with neoadjuvant therapy.

		DFS (*N* = 376)			OS (*N* = 376)		
Variables	No. of cases	No. of events	HR (95% CI)	*p*	No. of events	HR (95% CI)	*p*
Location of tumor							
Gastroesophageal junction	150	70			–		
Stomach	226	70	0.78 (0.55–1.09)	0.15	–	–	–
ypT stage							
ypT0‐T2	135	18			14		
ypT3‐T4	241	122	1.99 (1.09–3.45)	0.025	84	1.48 (0.79–2.78)	0.22
Vascular or lymphatic invasion							
No	231	55			37		
Yes	145	85	1.48 (1.01–2.18)	0.046	61	1.37 (0.87–2.15)	0.18
Venous invasion							
No	196	50			30		
Yes	180	90	0.76 (0.51–1.15)	0.19	68	1.13 (0.69–1.87)	0.63
Groups							
TRG0N0	48	3		<0.001	1		< 0.001
TRG1‐3N0	120	20	1.62 (0.45–5.86)	0.47	13	3.31 (0.41–26.52)	0.26
TRG0‐1N+	28	8	3.01 (0.75–12.18)	0.12	7	9.69 (1.15–81.98)	0.037
TRG2‐3N+	180	109	5.70 (1.57–20.73)	0.008	77	12.13 (1.52–96.88)	0.019
Neoadjuvant regimens							
ICI‐containing regimens	69	12		0.24	–		–
Platin‐based doublet regimens	193	79	1.05 (0.56–1.99)	0.87	–	–	–
Taxol‐based double or triplet regimens	94	47	1.32 (0.69–2.55)	0.41	–	–	–
Anti‐HER‐2 regimens	20	2	0.36 (0.081–1.65)	0.19	–	–	–

Abbreviations: CI, confidence interval; DFS, disease free survival; HER‐2, human epidermal growth factor receptor 2; HR, hazard ratio; ICI, immune checkpoint inhibitors; OS, overall survival; TRG, tumor regression grade; ypT, post‐therapy infiltration depth.

### 3.4 TRG combined with ypN status is significantly associated with different preoperative treatments

The chi‐squared test was performed to compare four types of neoadjuvant regimens among the different groups. We could generalize considerable discrepancies in pathological response among the regimens (*p* < 0.001), as outlined in Table S1. Patients undergoing immunotherapy had the largest proportion in achieving TRG0N0. There were 22 cases (31.9%) treated by ICI‐containing regimens, 17 cases (8.8%) accepting platin‐based doublet regimens, 6 cases (6.4%) undergoing taxol‐based doublet or triplet regimens, and 3 cases (15.0%) receiving anti‐HER‐2 regimens achieving TRG0N0, respectively. The TRG0N0 rate plus TRG1‐3N0 rate in patients treated with ICI‐containing regimens was 59.4%; this percentage was obviously higher than that in patients undergoing platin‐based doublet regimen (42.0%) and taxol‐based doublet or triplet regimens (35.1%). Due to the small number of patients, the results of this section should be interpreted with caution.

## 4 DISCUSSION

Our study aimed to assess the predictive value of combining TRG and ypN status as a prognostic indicator for patients with resectable, locally advanced GEJ, and GC who received NAT. After NAT, patients in the TRG0N0 group had the longest survival, followed by patients in the TRG1‐3N0, and TRG0‐3N+ groups. Among the patients in the TRG0‐3N+ group, the prognosis of those with TRG 0‐1 was better than that of those with TRG 2‐3. To the best of our knowledge, this is the first study to assess the combination of TRG and ypN status for GEJ and GC. The study is anticipated to shed light on a novel option for defining the endpoints in clinical trials.

Patients achieving pCR had a superior survival benefit, which is consistent with the finding of Li Ziyu for locally advanced GEJ and GC in a meta‐analysis.^17^ However, the proportion of patients achieving pCR was relatively small. There is still no consensus on how to perform postoperative adjuvant therapy for patients with different effects of NAT. Patients who respond well to preoperative treatment generally continue postoperative adjuvant chemotherapy according to the perioperative treatment mode. However, patients who derive limited benefits from NAT are recommended to receive multidisciplinary team (MDT) consultation. Future randomized trial can determine whether changing or intensifying treatment of non‐pCR will result in improvements in OS for these patients.^10^ Moreover, we need to analyze in‐depth the molecular biology characteristics of this group of people, hoping to find suitable treatment methods. More patients who could benefit from NAT were selected based on the evaluation of TRG and ypN status. TRG was first used to determine the efficacy of esophageal cancer after concurrent chemoradiotherapy.^18^ Thereafter, TRG was gradually applied to other malignant tumors including GC. Several TRG systems have been established by different investigators, but no agreement was reached on which one is the most representative for assessment of prognosis. Becker TRG system and Mandard system are the commonly used systems in clinical practice and have been evaluated in many studies.^10^, ^19^ The CSCO guidelines recommend TRG which is proposed by the 8th AJCC TNM classification or the NCCN guidelines.^8^ TRG0 using the NCCN definition would be TRG0‐ypN0. The predictive value of the NCCN TRG system on GEJ and GC had been recently proven. Sinnamon et al. have found that TRG using the contemporary NCCN definition is associated with OS in locoregional GC.^20^ In our study, not only the NCCN TRG system was evaluated, but also the relationship between NCCN TRG combined with ypN status and prognosis on GEJ and GC was revealed for the first time. Based on our results, TRG combined with ypN status was identified as an independent prognostic factor for DFS and OS. Furthermore, patients were subdivided into four groups, including pCR and TRG0N+, enabling a more comprehensive analysis than the simple division of patients into pCR and non‐pCR groups.

Patients in the TRG0N0 group achieved the longest survival, and patients with ypN+ had poorer outcomes than those with ypN0 for the rest patients. This result is consistent with those of previous studies. Smyth found that both TRG and ypN status were related to survival. However, only ypN status was revealed to function as an independent predictive factor of OS for patients with chemotherapy plus resection in the MAGIC trial. TRG was not identified as an independent marker for prognosis in the trial.^10^ Xu Xing drew a similar conclusion that TRG was not an independent prognostic predictor of GC.^21^ Donohoe Claire L. also put forward that none of the existing TRG measures had independent significance for the prognosis of esophageal and GEJ cancers.^22^ However, these researchers only concentrated on the prognostic significance of TRG or ypN status separately. According to Smyth, the mOS for patients with Mandard TRG 1‐2 was 17.3 months in the ypN+ group, which was longer than the 15.5 months found for patients with Mandard TRG 3‐5.^10^ Therefore, we proposed a novel evaluation approach of combining TRG with ypN status. A few studies reported that the combination of TRG and ypN status had a significant association with survival for esophageal carcinoma.^19^, ^23^, ^24^ But no such studies had been conducted for GEJ and GC patients. In the present study, the combination of TRG and ypN status was recognized to be of great prognostic value for GEJ and GC.

Both TRG and ypN status have been identified as short‐term efficacy evaluation markers of NAT; however, the association between them had not been demonstrated. At the end of our research, we made a supplement to this issue: patients undergoing ICI therapy had the greatest superiority in achieving TRG0N0; and the TRG0N0 rate plus TRG1‐3N0 rate in patients treated by ICI‐containing regimens was higher than that in patients undergoing chemotherapy only. Neoadjuvant chemotherapy is considered a standard modality for locally advanced GEJ or GC as it is recommended by multiple guidelines.^6^, ^7^, ^8^ In recent years, immunotherapy has become the standard first‐line therapy for GEJ and GC. The CheckMate 649 trial suggested that nivolumab with chemotherapy as the first‐line treatment significantly prolonged OS and DFS compared with chemotherapy in PD‐L1 CPS ≥5 patients.^25^ The ATTRACTION‐4 trial provided evidence that nivolumab combined with chemotherapy might potentially be a new approach for untreated, advanced GEJ, or GC.^26^ Besides, in the CheckMate 577 study, the adjuvant therapy of nivolumab was found to improve the mDFS from 11.0 to 22.4 months in comparison with the placebo, whose participants were esophageal or GEJ cancer without pCR after neoadjuvant chemoradiation.^27^ In the neoadjuvant setting, ICI is deemed to eradicate micrometastases and thus contribute to improved survival by inducing the activation of the immunologic system.^28^, ^29^, ^30^ Neoadjuvant immunotherapy for esophageal cancer has shown promising initial results. A retrospective analysis revealed that neoadjuvant immunotherapy plus chemotherapy for patients with locally advanced esophageal squamous cell carcinoma led to an advantage in pathological response, and could prolong DFS compared with chemotherapy alone.^31^ Recently, two single‐arm clinical trials with small sample sizes suggested that neoadjuvant immunotherapy could improve the pCR rate of GC patients.^32^, ^33^ After signing written informed consents by some GC patients, who might potentially benefit from ICI, or their legal guardians, we made exploratory attempts to treat these patients with ICI before operation in clinical practice. Encouragingly, our retrospective study revealed that this modality had the highest TRG0N0 (pCR) rate, symbolizing superior survivals. This study was the first time to illustrate the short‐term therapeutic effect of ICI versus chemotherapy alone as NAT for GEJ and GC, confirming the confidence that NAT containing ICI would lead to successful outcomes. The results also highlight the potential of TRG combined with ypN status as a surrogate endpoint for neoadjuvant clinical trials.

Despite of the promising results, our study had some limitations. This study was a single‐center retrospective exploratory analysis that has not been validated. Furthermore, although patients meeting the TRG0N+ criteria were eligible for inclusion in this study, only three such patients were enrolled. As a result, the conclusion was underpowered for application to the subgroup. Finally, the follow‐up time for patients receiving neoadjuvant immunotherapy was short, warranting long‐term survival validation.

## 5 CONCLUSIONS

Based on our results, TRG combined with ypN status is a novel independent predictor of both DFS and OS in resectable, locally advanced GEJ, and GC. The application of neoadjuvant immunotherapy increased the TRG0N0 and TRG1‐3N0 rates, which indicated a good prognosis in our findings. However, prospective and multicenter studies with a larger number of participants are required to verify the results.

## AUTHOR CONTRIBUTIONS

**Hongyan Yin:** Data curation (equal); formal analysis (equal); investigation (equal); software (equal); writing – original draft (equal). **Qian Yao:** Data curation (equal); formal analysis (equal); methodology (equal). **Yi Xie:** Data curation (equal); validation (equal); writing – review and editing (equal). **Dongfeng Niu:** Validation (equal). **Wenyan Jiang:** Validation (equal). **Huiying Cao:** Supervision (equal). **Xujiao Feng:** Writing – original draft (supporting). **Yanyan Li:** Methodology (supporting); writing – review and editing (supporting). **Yilin Li:** Writing – review and editing (equal). **Xiaotian Zhang:** Funding acquisition (equal); writing – review and editing (equal). **Lin Shen:** Funding acquisition (equal); investigation (equal); resources (equal); visualization (equal). **Yang Chen:** Conceptualization (equal); formal analysis (equal); funding acquisition (equal); investigation (equal); project administration (equal); supervision (equal); visualization (equal); writing – review and editing (equal).

## FUNDING INFORMATION

This work was supported by the Beijing Natural Science Foundation, grant number 7222021 (Y.C.), Z200015 (XT.Z.); National Natural Science Foundation of China, grant number 82203881 (Y.C.), 91959205 (L.S.), 82272627 (XT.Z.); Science Foundation of Peking University Cancer Hospital, XKFZ2303 (L.S.); Beijing Hospitals Authority Youth Programme, code [QML20231115], Clinical Medicine Plus X‐Young Scholars Project of Peking University (PKU2023LCXQ041).

## CONFLICT OF INTEREST STATEMENT

The authors have no conflict of interest.

## ETHICS STATEMENT

Approval of the research protocol by an Institutional Reviewer Board: The study was conducted in accordance with the Declaration of Helsinki and approved by the Institutional Review Board of Peking University Cancer Hospital (protocol code 2020KT08).

## Supporting information


**Data S1.** Supporting information

## Data Availability

The datasets used and/or analyzed during the current study are available from the corresponding author on reasonable request.
