# Supplementary material for: Tumor regression grade combined with post‐therapy lymph node status: A novel independent prognostic factor for patients treated with neoadjuvant therapy followed by surgery in locally advanced gastroesophageal junction and gastric carcinoma
Source: Cancer Med. 2023 Sep 25;12(19):19633–43. doi: 10.1002/cam4.6597 (PMC10587920; doi:10.1002/cam4.6597)
Supplement: Supplementary file 1 — Data S1. Supporting information [file CAM4-12-19633-s001.doc]

**Tumor regression grade combined with post-therapy lymph node status: A novel independent prognostic factor for patients treated with neoadjuvant therapy followed by surgery in locally advanced gastroesophageal junction and gastric carcinoma**

Hongyan Yin^1†, 2^, Qian Yao^3†^, Yi Xie^1†^, Dongfeng Niu^3^, Wenya Jiang^2^, Huiying Cao^2^, Xujiao Feng^1^, Yanyan Li^1^, Yilin Li^1^, Xiaotian Zhang^1^, Lin Shen^1*^ and Yang Chen^1*^

^1^ Department of Gastrointestinal Oncology, Key Laboratory of Carcinogenesis and Translational Research (Ministry of Education), Peking University Cancer Hospital and Institute, Beijing, China

^2^ Department of Gastroenterology, CANGZHOU People’s Hospital, Hebei, China

^3^ Department of Pathology, Key Laboratory of Carcinogenesis and Translational Research (Ministry of Education), Peking University Cancer Hospital and Institute, Beijing, China

† These authors contributed equally: Hongyan Yin, Qian Yao, Yi Xie

***** Correspondence: shenlin@bjmu.edu.cn (L.S.), Yang_chen@bjcancer.org (Y.C.); Tel.: 010-88196747

**
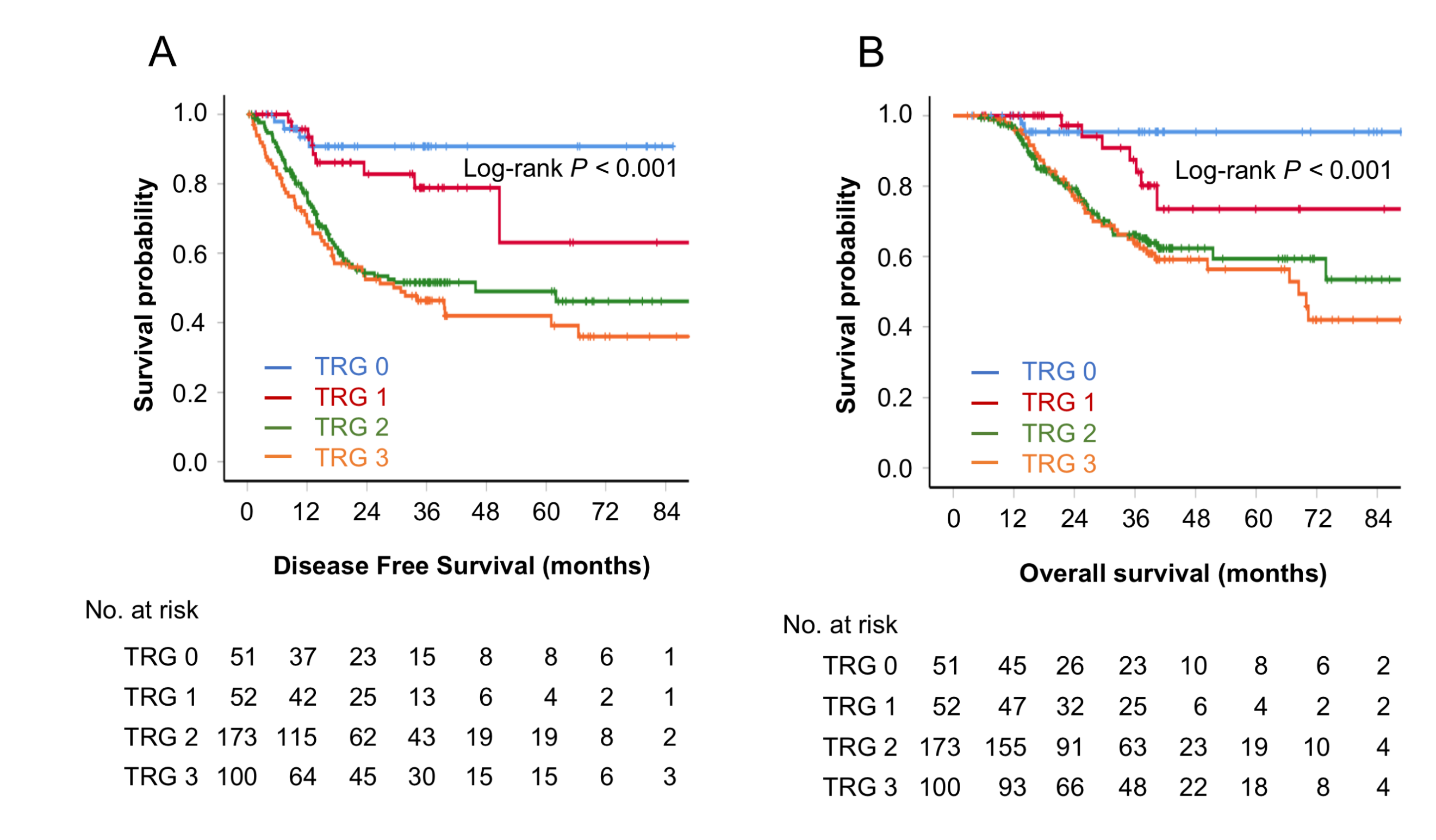
**

**Fig. S1.** Kaplan-Meier curves of disease-free survival (A) and overall survival (B) based on tumor regression grade (TRG).

**
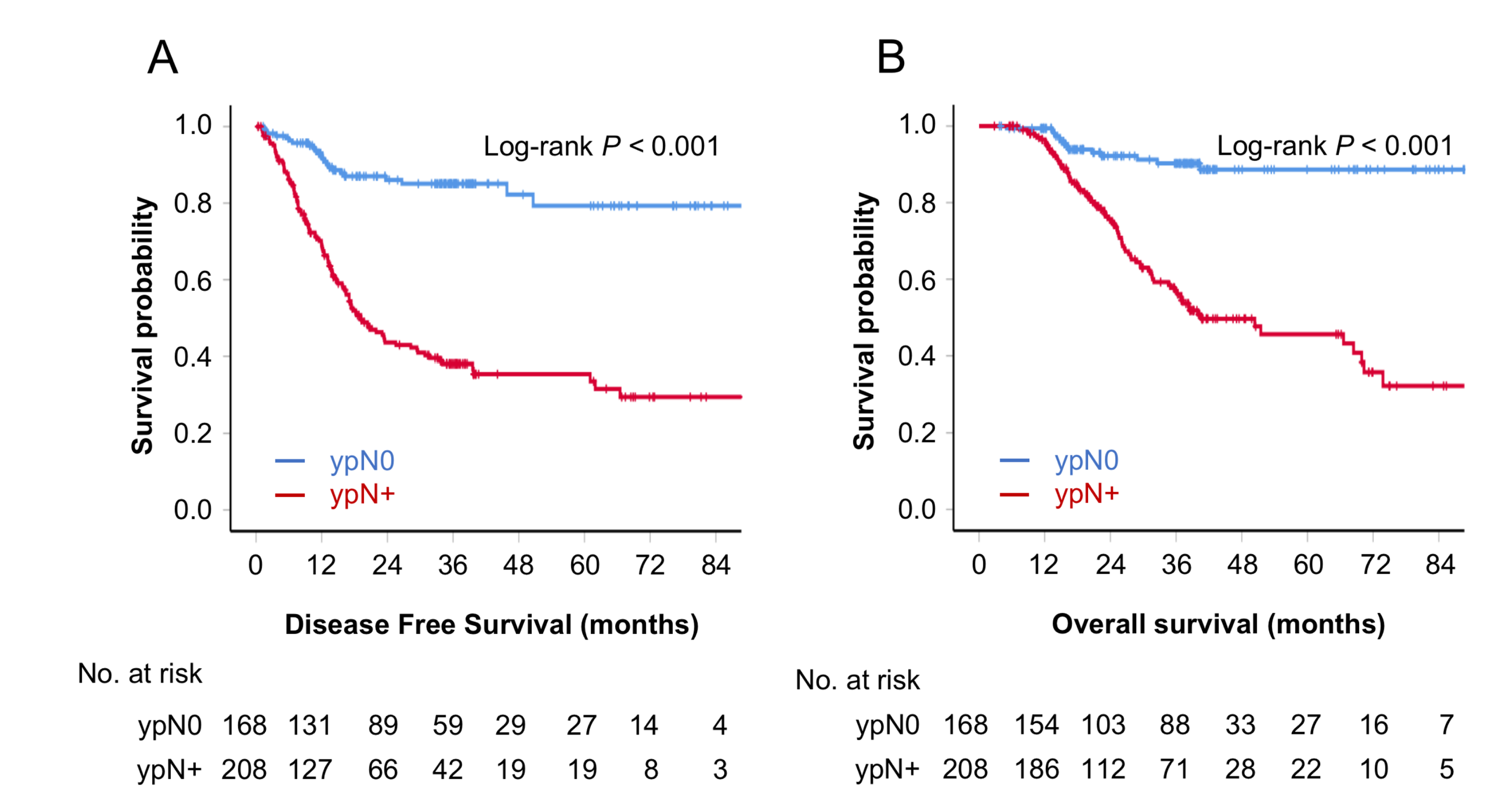
**

**Fig. S2.** Kaplan-Meier curves of disease-free survival (A) and overall survival (B) according to post-therapy lymph node (ypN) status: ypN-negative (ypN0) and ypN-positive (ypN+) groups.

**
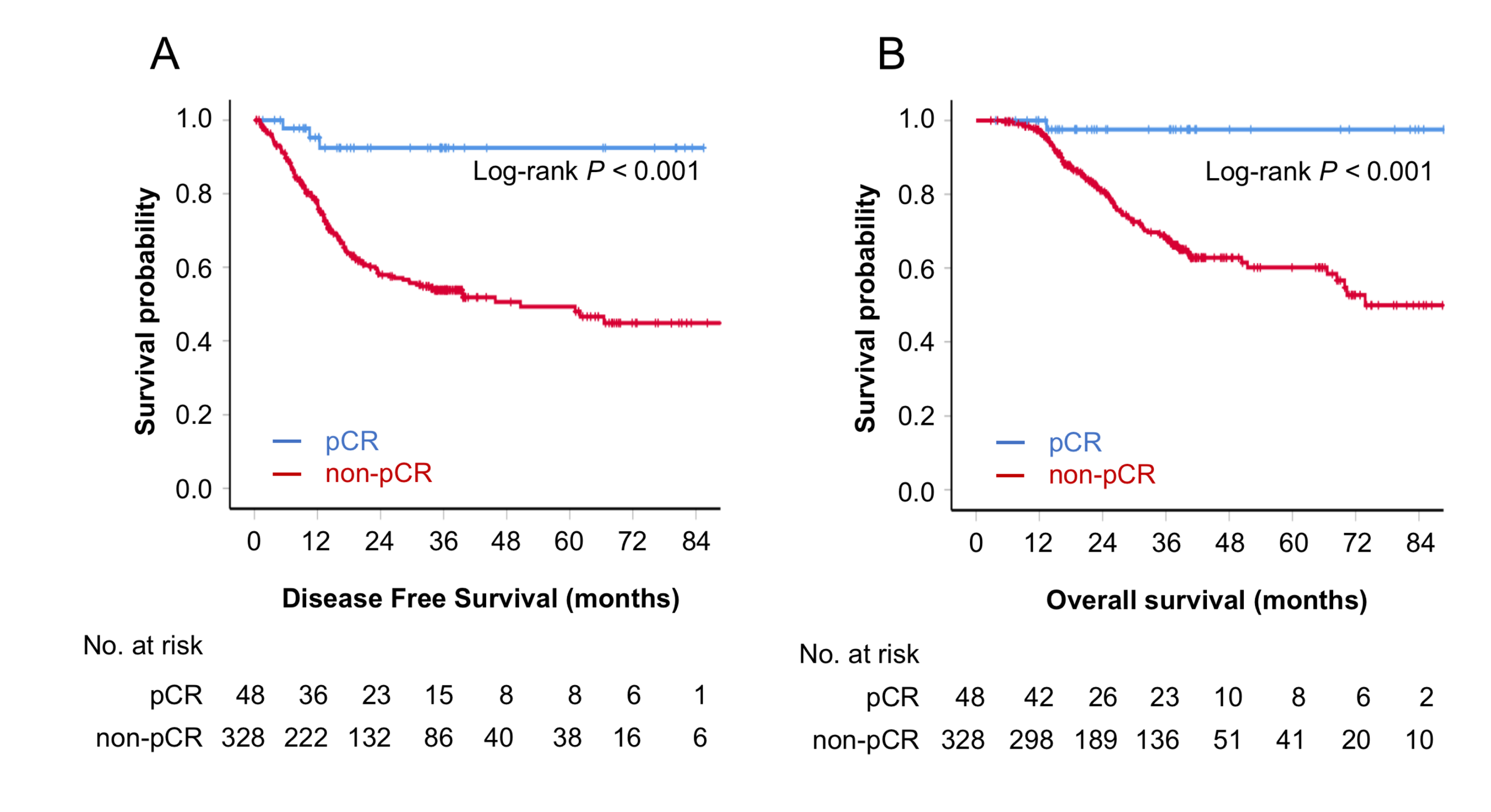
**

**Fig. S3.** Kaplan-Meier curves of disease-free survival (A) and overall survival (B) according to pathological complete response (pCR) or non-pCR.

**
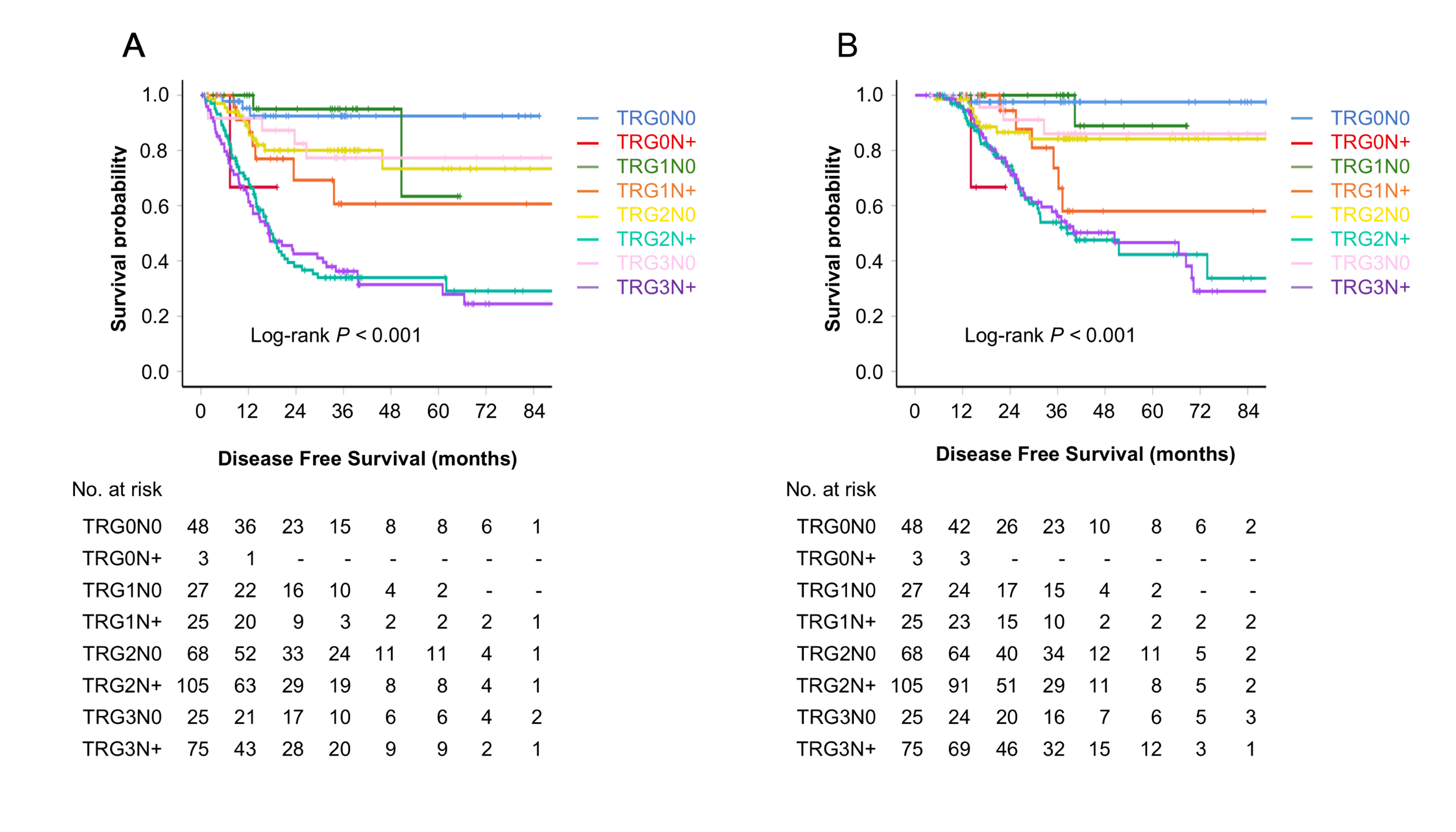
**

**Fig. S4.** Kaplan-Meier curves of disease-free survival (A) and overall survival (B) according to tumor regression grade (TRG) and post-therapy lymph node (ypN) status: TRG0N0, TRG0N+, TRG1N0, TRG1N+, TRG2N0, TRG2N+, TRG3N0, TRG3N+ groups.

**Table S1. Association of different neoadjuvant regimens with different groups**

| Groups | No. of  cases | ICI-based regimens | Platin-based doublet regimens | Taxol-based doublet or triplet regimens | Anti-HER-2  regimens | P |
| --- | --- | --- | --- | --- | --- | --- |
| TRG0N0 | 48 | 22 (31.9%) | 17 (8.8%) | 6 (6.4%) | 3 (15.0%) | < 0.001 |
| TRG1-3N0 | 120 | 19 (27.5%) | 64 (33.2%) | 27 (28.7%) | 10 (50.0%) |  |
| TRG0-1N+ | 28 | 4 (5.8%) | 14 (7.3%) | 9 (9.6%) | 1 (5.0%) |  |
| TRG2-3N+ | 180 | 24 (34.8%) | 98 (50.8%) | 52 (55.3%) | 6 (30.0%) |  |

Abbreviations: TRG, tumor regression grade; ICI, immune checkpoint inhibitors; HER-2, human epidermal growth factor receptor 2.
